# Epidemiology and risk factors of oral frailty among older people: an observational study from China

**DOI:** 10.1186/s12903-024-04149-1

**Published:** 2024-03-21

**Authors:** Yueheng Yin, Yue Zhao, Yang Fei, Ying Liu, Yun Ji, Enfang Shan, Shuzhen Niu, Ying Xing, Jingjing Ding, XianWen Li

**Affiliations:** 1 School of Nursing, Nanjing Medical University, No.101 Longmian Avenue, Nanjing, 211166 Jiangsu China; 2 Chunhua Community Health Service Centre, Nanjing, 211166 Jiangsu China; 3 Sir Run Run Hospital of Nanjing Medical University, Nanjing, 211166 Jiangsu China

**Keywords:** Oral frailty, Prevalence, Risk factor, Measurement method

## Abstract

**Objectives:**

This study aimed to compare the prevalence of oral frailty among community-dwelling older people in Nanjing, China with the usage of different measurements, and to investigate the potential risk factors of oral frailty.

**Design:**

Cross-sectional study.

**Setting and participants:**

A total of 338 community-dwelling older people in Nanjing, China were recruited.

**Methods:**

Oral frailty was measured based on the Oral Frailty Index-8 (OFI-8) scale and other measurement methods including the number of natural teeth (TN), repetitive saliva-swallowing test (RSST), and oral diadochokinesis (ODK). The chi-square test and the binary logistic regression analysis were performed to identify potential risk factors for oral frailty.

**Results:**

There were 310 participants included in the analysis. Prevalence of oral frailty by using the OFI-8, OFI-8 + TN, OFI-8 + ODK, OFI-8 + TN + ODK and RSST measurement methods were 69.0%, 27.4%, 51.9%, 21.0% and 2.9%, respectively. Passive smoking (OR = 2.04; 95%CI 1.03–4.03), being widowed/unmarried (OR1 = 2.53; 95%CI 1.25–5.10; OR2 = 2.94; 95%CI 1.12–7.77), pre-frailty (OR = 1.76; 95%CI 1.03–3.01), frailty (OR = 3.01; 95%CI 1.39–6.54), and aged 80 years and above (OR = 3.99; 95%CI 1.35–11.81) were found to be risk factors of oral frailty by the usage of the four kinds of measurement methods.

**Conclusions and implications:**

The definition and diagnostic criteria of oral frailty are strongly needed to be unified in future research. Only subjective assessment is not enough for assessing oral frailty. Among objective indicators, RSST is not suitable as a screening method for oral frailty. In addition, objective indicators including TN and ODK should be valued for early screening and preventive interventions. The risk factors of oral frailty include physical frailty, passive smoking, and being widowed.

**Supplementary Information:**

The online version contains supplementary material available at 10.1186/s12903-024-04149-1.

## Introduction

With the aging of the population, oral health issues among the older people are becoming increasingly prominent. Oral frailty is a novel concept proposed in recent years, which is defined as age-related decline of oral function and driven by a series of dysfunction that deteriorate oral health [1,2]. The decline of age-related oral function includes loss of teeth, difficulty in chewing or swallowing, oropharyngeal dysphagia, poor oral hygiene, etc. [2]. Compared with a single oral health indicator such as the teeth number or chewing ability, oral frailty is more reflective of overall oral function and its trend. Oral frailty is found to be associated with both physical and psychological problems [3]. A set of physical problems such as eating disorders, malnutrition, sarcopenia, disability, and even death was found to be related to oral frailty [4]. A systematic review of five longitudinal studies from three countries reported people with oral health problems have a much higher risk of being physically frail [5]. A cross-sectional study of 682 community-dwelling older adults reported significant associations between oral frailty and declines in social function [6]. Older people’s oral health status is significant since it reflects a multidimensional senile symptom.

Oral frailty accounts for a large proportion of the older population. In previous studies, the prevalence of oral frailty among older people was reported to be 14% [7] or even 44.7% [8], which may be caused by the one-sided assessment tools they selected. For instance, Nagatani’s et al. study [9] used six components including the number of remaining teeth, masticatory status, tongue pressure, oral motor skills, and subjective difficulties in eating and swallowing to identify oral frailty, but lacking assessments of oral health-related behaviours and social participation. Kusunoki et al. [10] used oral frailty index-8 (OFI-8) as the tool for screening oral frailty, but lacking objective indicators. The OFI-8 scale, a tool proposed to help screen older adults at risk of oral frailty, is with good validity [1]. The OFI-8 includes the most important indicators of oral frailty such as the false tooth usage and chewing ability, and it also includes the assessments of oral health-related behaviours and social participation. Due to its convenience and comprehensiveness, the OFI-8 scale is frequently used to identify oral frailty [10,11].

At present, there is no consensus on the diagnosis and measurement methods of oral frailty [2]. Yang et al. concluded four attributes of oral frailty including hypofunction, predisposing in nature, non-specific criteria and multidimensional through concept analysis [12]. In addition, Kugimiya et al. found that oral frailty was accompanied by a decrease in mental and physical functions, suggesting that oral frailty should be identified with a multidimensional approach [12,13]. Currently, a limit of epidemiological research on oral frailty among the older population could be found. There was no enough data to get the conclusion of the best measurement criteria for oral frailty. Even though the OFI-8 scale [8,14] is used by several studies, other objective measurement indicators such as the number of natural teeth, oral diadochokinesis (ODK), and repeated saliva swallowing tests (RSST) [15] should also be considered. Hence, we are interested in the prevalence of oral frailty with the combination of subjective and objective measurements.

To the best of our knowledge, no study has yet investigated the prevalence of oral frailty by applying different measurements. In addition, the potential risk factors related to oral frailty are worth to be explored, which can help prevent the incidence. Comorbidity, smoking, alcohol drinking, and physical function are related to single oral condition of older people [16–18]. But the risk factors of comprehensive oral status, i.e., oral frailty, are still not clear. Therefore, in this study, we aimed to compare the prevalence of oral frailty among community-dwelling older people in Nanjing, China with the usage of different measurements such as the OFI-8, the number of natural teeth, oral diadochokinesis (ODK), and repeated saliva swallowing tests (RSST), and to investigate the potential risk factors of oral frailty.

## Methods

### Study design

A cross-sectional study was conducted, and reported by following the Strengthening the Reporting of Observational Studies (STROBE) statement [19]. Our study was part of *Nanjing Brain Health Cohort*, a prospective cohort study using a multistage sampling method to assess and recognize risk factors related to speech and oral frailty for cognitive impairment. For this study, we selected two community hospitals randomly in Jiangning District, Nanjing. Participants were recruited via general practitioners and nurses in community hospitals. Eligible participants were invited to a quiet room in the community hospitals where the questionnaire and test took place. Data was collected by research assistants who were professionally trained.

### Setting and participants

The study was conducted in two community health centers in Nanjing, the capital city of Jiangsu Province in the east of China, from June to August 2023. Community-dwelling individuals aged 60 years and above who have lived in the community for over 6 months were included. Those who could not communicate or with severe cognitive impairment were excluded. Severe cognitive impairment was defined based on the Clinical Dementia Rating (CDR), which is a standardized tool used to assess the severity of dementia [20]. A score of more than 1 point was rated as severe cognitive impairment. In addition, participants who were diagnosed with Parkinson’s disease, cerebrovascular disease, epilepsy, Alzheimer’s disease, brain trauma, brain tumors, encephalitis, and schizophrenia were excluded. For the sample size calculation, considering the prevalence of oral frailty as 14.4 [3], we set α as 5%, allowable error as 0.086 and dropout rate as 20% in PASS V.15 (NCSS, Kaysville, Utah, USA). The sample size was 348.

### Measures

Oral frailty was measured based on the OFI-8 scale and extra objective measurements, which included the number of natural teeth (TN), repetitive saliva-swallowing test (RSST), and oral diadochokinesis (ODK).

1. The OFI-8 [1,21] scale assesses oral health-related behaviours and potential indicators of oral frailty from 5 dimensions, which include false tooth, swallowing ability, chewing ability, oral health-related behaviours, and social participation. The questionnaire consists of eight items: Q1. Do you have any difficulties eating tough foods compared to 6 months ago? Q2. Have you choked on your tea or soup recently? Q3. Do you use dentures? Q4. Do you often have a dry mouth? Q5. Do you go out less frequently than you did last year? Q6. Can you eat hard foods like squid jerky or pickled radish? Q7. How many times do you brush your teeth in a day? (3 or more times/day), and Q8. Do you visit a dental clinic at least annually? Using the standard protocol, if subjects answered “yes” to Items 1, 2, or 3, two points were given for each answer. If the subjects answered “yes” to Items 4 and 5, one point was given for each answer. If the subjects answered “no” to Items 6, 7, or 8, one point was given for each answer. The screening criterion was defined as the sum of the scores called OFI-8 score. The total OFI-8 score ranges from 0 to 11 points, with higher scores indicating poorer oral health. An OFI-8 score over 4 points was defined as oral frailty, which has tested its high sensitivity and specificity. The Cronbach’s α coefficient is 0.692.
2. The TN was evaluated by a trained dentist. Fewer than 20 natural teeth were considered to be one item of oral frailty [1].
3. The RSST was used to screen the decline in swallowing function [22]. Participants were asked to perform repetitive voluntary swallowing as quickly as possible for 30 s, and the swallowing count during this time was used as the RSST score. Less than 3 times swallowing saliva/30 sec was considered as a kind of performance of oral frailty.
4. Oral diadochokinesis (‘pa’, ‘ta’, and ‘ka’ times/sec) was assessed to evaluate the function of the lips, the tip of the tongue and the posterior region of the tongue [23]. Participants were asked to articulate each syllable 20 times repetitively. Articulation counts were measured using the timing function of the phone. The number of repetitions/second was calculated separately for each syllable. ODK /pa/ (men: <5.2 times/s, women: <5.6 times/s), /ta/ (men: <5.2 times/s, women: <5.4 times/s) and /ka/ (men: <4.4 times/s, women: <5.0 times/s) were used to assess oral frailty [1].

### Covariates

Demographic data including age, sex, education level, marriage, employment status, living arrangement, source of income, and monthly income were recorded through face-to-face questionnaire investigation. The health-related information included self-reported hypertension, diabetes, coronary heart disease, gastritis, rheumatoid arthritis, frailty and depression. Depressive symptoms were evaluated by the 5-item Geriatric Depression Scale (GDS-5) [24]. The frailty phenotype scale by Fried et al. [25] was used to assess frailty. Lifestyle factors were also collected which included alcohol drinking, smoking, passive smoking, sedentary times, tea-drinking, and leisure activity [26].

### Statistical analysis

All statistical analyses were performed by using the SPSS 27.0 (IBM Corporation, Armonk, NY, USA). Missing values associated with oral frailty were eliminated. Missing categorical variables were filled with modes. Continuous variables are presented as mean and standard deviation (SD), or median and interquartile range (IQR) based on the distribution of the quantitative variables. Categorical variables are presented as frequencies and percentages. The chi-square test was performed to identify variables associated with the presence of any oral frailty (OF) group. A cut point of *P* < 0.20 was used to determine which variables were included in subsequent analysis. The binary logistic regression analyses were then used to identify potential independent risk factors for oral frailty. The odds ratios (ORs) were computed by using logistic regression and adjusted for sex, gender, and education, since the variables have been demonstrated to be strongly associated with oral frailty [1,27,28]. The Hosmer-Lemeshow test was used to check the fit of the model. The sensitivity analysis, in which outliers were not deleted, was used to test the stability of the risk factors for oral frailty. All statistical analyses were two-tailed, and statistical significance was set as *P* < 0.05.

## Results

A total of 351 older people from the community were investigated. In the screening stage, 7 people did not meet the inclusion criteria and 6 people did not complete the whole investigation, which left 338 participants being approached. Due to the outliers were found from 28 participants, eventually, 310 participants were included in the data analysis.

### Characteristics of the participants

The characteristics of the participants (*n* = 310) are shown in Table 1. The age of the participants was 70.0 (66.0, 74.0) years old, and the majority (50.6%) were from 70 to 79 years old. A slight majority was female (51.6%). Most participants received primary school education (45.2%). Most of them lived with their spouses (67.1%) and relied on government subsidies (50.6%). 61.9% of the participants showed low leisure activities. 68.4% of the participants had chronic diseases, among which hypertension was the most common (59.0%).

**Table 1:** Sociodemographic and medical characteristics of all participants (*N* = 310)

Variables	N (%)
Sociodemographic	
Age (years)	70.0 (66.0, 74.0)
Age group (years)	
60–69	132 (42.6)
70–79	157 (50.6)
80+	21 (6.8)
Sex	
> Female	160 (51.6)
Male	150 (48.4)
Education level	
Illiteracy	61 (19.7)
Primary school	140 (45.2)
Middle school	69 (22.2)
High school and above	40 (12.9)
Marriage	
Married	258 (83.2)
Widowed/unmarried	52 (16.8)
Employment status	
Employed	31 (10.0)
Retired	240 (77.4)
Others	39 (12.6)
Living arrangement	
Living alone	60 (19.4)
With spouse	208 (67.1)
With children	31 (10.0)
Others	11 (3.5)
Source of income	
Pension	91 (29.4)
Child support or others	62 (20.0)
Government subsidy	157 (50.6)
Monthly incomes	
<2000	217 (70.0)
2000–4000	48 (15.5)
>4000	45 (14.5)
**Lifestyle**	
Alcohol drinking	62 (20.0)
Sedentary time	
<5 h/d	218 (70.3)
5 ~ 8 h/d	76 (24.5)
≥ 8 h/d	16 (5.2)
Smoking	59 (19.0)
Passive smoking	73 (23.5)
Tea drinking	113 (36.5)
Leisure activities	
Low	192 (61.9)
Moderate	40 (12.9)
High	78 (25.2)
**Comorbidities**	
Chronic disease	212 (68.4)
Hypertension	183 (59.0)
Coronary heart disease	26 (8.4)
Rheumatoid arthritis	9 (2.9)
Diabetes	64 (20.6)
Gastritis	16 (5.2)
Frailty	
Robustness	132 (42.6)
Pre-frailty	130 (41.9)
Frailty	48 (15.5)
Depression	77 (24.8)

*Note* Continuous variable without a normal distribution is presented as median and interquartile range

Categorical variables are presented as n (%)

### Prevalence of oral frailty based on different measurement methods

The prevalence of oral frailty according to the 5 different measurement methods are presented in Table 2. Since the number of older people screened by the OFI-8 + RSST measurement method was only 6, we do not show the results here. Prevalence rates by using the OFI-8, OFI-8 + TN, OFI-8 + ODK, OFI-8 + TN + ODK and RSST measurement methods were 69.0%, 27.4%, 51.9%, 21.0% and 2.9%, respectively. According to the OFI-8 + TN measurement method, the analysis showed a significant difference in the prevalence of oral frailty based on age group (*P* = 0.01), sex (*P* = 0.045), marriage (*P* = 0.022), source of income (*P* = 0.038). With the OFI-8 + ODK measurement method, sedentary time (*P* = 0.013), rheumatoid arthritis (*P* = 0.038), diabetes (*P* = 0.014) and physical frailty (*P* = 0.019) were found to have statistically significant differences. In addition, age group (*P* = 0.006), marriage (*P* = 0.023) and rheumatoid arthritis (*P* = 0.022) were found to have statistically significant differences in terms of the OFI-8 + TN + ODK measurement method.

**Table 2:** Prevalence of oral frailty by different measurement methods (*N* = 310)

Variables	OFI-8	^χ2^	P^*^	OFI-8 + TN	^χ2^	P^*^	OFI-8 + ODK	^χ2^	P*	OFI-8 + TN + ODK	^χ2^	P^*^	RSST	^χ2^	P^*^
	n (%)			n (%)			n (%)			n (%)			n (%)		
Sociodemographic															
Age (years)	70.0 (67.0, 74.0)			72.60 ± 6.09			71.0 (67.0, 75.0)			72.0 (68.5, 77.0)			70.78 ± 4.18		
Age group (years)															
60–69	90 (29.0)	0.11	0.95	25 (8.1)	9.31	**0.010**	68 (21.9)	3.53	**0.17**	19 (6.1)	10.15	**0.006**	3 (1)	0.68	0.75^‡^
70–79	109 (35.2)			51 (16.5)			78 (25.2)			37 (11.9)			6 (1.9)		
80+	15 (4.8)			9 (2.9)			15 (4.8)			9 (2.9)			0 (0)		
Sex															
female	108 (34.8)	0.36	0.55	36 (11.6)	4.02	**0.045**	90 (29.0)	2.47	**0.12**	32 (10.3)	0.19	0.67	3 (1)	-	0.32^†^
male	106 (34.2)			49 (15.8)			71 (22.9)			33 (10.6)			6 (1.9)		
Education level															
illiteracy	40 (12.9)	3.16	0.37	16 (5.2)	1.55	0.67	31 (10.0)	2.33	0.51	14 (4.5)	1.52	0.68	1 (0.3)	1.43	0.76^‡^
primary school	103 (33.2)			43 (13.9)			79 (25.5)			32 (10.3)			6 (1.9)		
middle school	43 (13.9)			16 (5.2)			32 (10.3)			11 (3.5)			1 (0.3)		
high school and above	28 (9.0)			10 (3.2)			19 (6.1)			8 (2.6)			1 (0.3)		
Marriage															
married	174 (56.1)	1.82	**0.18**	64 (20.6)	5.28	**0.022**	130 (41.9)	1.48	0.22	48 (15.5)	5.18	**0.023**	7 (2.3)	-	0.65^†^
widowed/unmarried	40 (12.9)			21 (6.8)			31 (10.0)			17 (5.5)			2 (0.6)		
Employment status															
employed	17 (5.5)	4.92	**0.09**	5 (1.6)	5.68	**0.06**	12 (3.9)	4.49	**0.11**	4 (1.3)	3.52	**0.17**	2 (0.6)	3.35	0.14^‡^
retired	166 (53.5)			64 (20.6)			124 (40.0)			49 (15.8)			5 (1.6)		
others	31 (10.0)			16 (5.2)			25 (8.1)			12 (3.9)			2 (0.6)		
Living arrangement															
living alone	40 (12.9)	2.33	0.51	14 (4.5)	4.28	0.23	28 (9.0)	0.88	0.83	9 (2.9)	5.29	**0.15**	1 (0.3)	6.09	0.08^‡^
with spouse	141 (45.5)			56 (18.1)			110 (35.5)			43 (13.9)			6 (1.9)		
with children	25 (8.1)			13 (4.2)			17 (5.5)			11 (3.5)			0 (0)		
others	8 (2.6)			2 (0.6)			6 (1.9)			2 (0.6)			2 (0.6)		
Source of income															
pension	63 (20.3)	3.48	**0.18**	20 (6.5)	6.55	**0.038**	47 (15.2)	0.15	0.93	16 (5.2)	2.01	0.37	3 (1)	4.19	0.11^‡^
child support or others	37 (11.9)			12 (3.9)			31 (10.0)			11 (3.5)			4 (1.3)		
government subsidy	114 (36.8)			53 (17.1)			83 (26.8)			38 (12.3)			2 (0.6)		
Monthly incomes															
<2000	152 (49.0)	1.15	0.56	67 (21.6)	4.45	**0.11**	118 (38.1)	2.56	0.28	50 (16.1)	2.02	0.36	7 (2.3)	0.14	1^‡^
2000–4000	30 (9.7)			10 (3.2)			20 (6.5)			7 (2.3)			1 (0.3)		
>4000	32 (10.3)			8 (2.6)			23 (7.4)			8 (2.6)			1 (0.3)		
**Lifestyle**															
Alcohol drinking															
no	176 (56.8)	2.17	**0.14**	67 (21.6)	0.10	0.75	135 (43.5)	3.11	**0.08**	53 (17.1)	0.12	0.73	5 (1.6)	-	0.08^†^
yes	38 (12.3)			18 (5.8)			26 (8.4)			12 (3.9)			4 (1.3)		
Sedentary time															
<5 h/d	158 (51)	4.64	**0.10**	61 (19.7)	0.13	0.94	125 (40.3)	8.61	**0.013**	45 (14.5)	0.15	0.93	7 (2.3)	0.09	1^‡^
5 ~ 8 h/d	45 (14.5)			20 (6.5)			30 (9.7)			17 (5.5)			2 (0.6)		
≥ 8 h/d	11 (3.5)			4 (1.3)			6 (1.9)			3 (1.0)			0 (0)		
Smoking															
no	170 (54.8)	1.05	0.31	63 (20.3)	3.57	**0.06**	128 (41.3)	0.47	0.50	50 (16.1)	0.87	0.35	5 (1.6)	-	0.07^†^
yes	44 (14.2)			22 (7.1)			33 (10.6)			15 (4.8)			4 (1.3)		
Passive smoking															
no	157 (50.6)	3.66	**0.06**	61 (19.7)	1.43	0.23	117 (37.7)	2.66	**0.10**	45 (14.5)	2.38	**0.12**	7 (2.3)	-	1^†^
yes	57 (18.4)			24 (7.7)			44 (14.2)			20 (6.5)			2 (0.6)		
Tea drinking															
no	137 (44.2)	0.07	0.80	53 (17.1)	0.07	0.79	102 (32.9)	0.01	0.94	41 (13.2)	0.01	0.93	6 (1.9)	-	1^†^
yes	77 (24.8)			32 (10.3)			59 (19.0)			24 (7.7)			3 (1)		
Leisure activities															
low	128 (41.3)	1.45	0.49	58 (18.7)	2.11	0.35	93 (30.0)	3.11	0.21	44 (14.2)	1.96	0.38	6 (1.9)	0.15	1^‡^
moderate	30 (9.7)			10 (3.2)			21 (6.8)			9 (2.9)			1 (0.3)		
high	56 (18.1)			17 (5.5)			47 (15.2)			12 (3.9)			2 (0.6)		
**Comorbidities**															
Chronic disease															
no	65 (21)	0.49	0.48	32 (10.3)	1.97	**0.16**	44 (14.2)	2.84	**0.09**	23 (7.4)	0.54	0.46	4 (1.3)	-	0.47^†^
yes	149 (48.1)			53 (17.1)			117 (37.7)			42 (13.5)			5 (1.6)		
Hypertension															
no	82 (26.5)	2.01	**0.16**	38 (12.3)	0.68	0.41	59 (19.0)	2.59	**0.11**	29 (9.4)	0.45	0.50	5 (1.6)	-	0.50
yes	132 (42.6)			47 (15.2)			102 (32.9)			36 (11.6)			4 (1.3)		
Coronary heart disease															
no	193 (62.3)	1.83	**0.18**	76 (24.5)	0.74	0.39	146 (47.1)	0.38	0.54	58 (18.7)	0.61	0.44	7 (2.3)	-	0.17^†^
yes	21 (6.8)			9 (2.9)			15 (4.8)			7 (2.3)			2 (0.6)		
Rheumatoid arthritis															
no	206 (66.5)	-	0.28^†^	80 (25.8)	-	**0.07** ^**†**^	153 (49.4)	-	**0.038** ^**†**^	60 (19.4)	-	**0.022** ^**†**^	9 (2.9)	-	1^†^
yes	8 (2.6)			5 (1.6)			8 (2.6)			5 (1.6)			0 (0)		
Diabetes															
no	166 (53.5)	1.34	0.25	64 (20.6)	1.18	0.28	119 (38.4)	6.06	**0.014**	46 (14.8)	3.70	**0.054**	7 (2.3)	-	1^†^
yes	48 (15.5)			21 (6.8)			42 (13.5)			19 (6.1)			2 (0.6)		
Gastritis															
no	203 (65.5)	-	1.00^†^	81 (26.1)	-	1^†^	153 (49.4)	0.03	0.87	64 (20.6)	-	0.21	9 (2.9)	-	1^†^
yes	11 (3.5)			4 (1.3)			8 (2.6)			1 (0.3)			0 (0)		
Frailty															
robustness	84 (27.1)	4.29	**0.12**	40 (12.9)	1.15	0.56	58 (18.7)	7.93	**0.019**	28 (9.0)	0.19	0.91	6 (1.9)	1.98	0.39^‡^
pre-frailty	92 (29.7)			34 (11.0)			71 (22.9)			26 (8.4)			2 (0.6)		
frailty	38 (12.3)			11 (3.5)			32 (10.3)			11 (3.5)			1 (0.3)		
Depression															
no	161 (51.9)	0.002	0.97	61 (19.7)	0.72	0.40	117 (37.7)	1.11	0.29	45 (14.5)	1.55	0.21	7 (2.3)	-	1^†^
yes	53 (17.1)			24 (7.7)			44 (14.2)			20 (6.5)			2 (0.6)		
**Total**	214 (69.0)			85 (27.4)			161 (51.9)			65 (21.0)			9 (2.9)		

*Note* OF, Oral Frailty; OFI-8, Oral Frailty Index-8; TN, number of natural teeth; ODK, Oral diadochokinesis. Continuous variables with a normal distribution are shown as mean ± SD. Continuous variables without a normal distribution are presented as median and interquartile range. Values with *P* < 0.2 are bolded. The percentages in brackets are based on a total population of 310 participants. ^*^ Chi-squared test; ^†^ Fisher’s exact test; ^‡^ Fisher-Freeman-Halton exact test

### Risk factors for oral frailty

Variables associated with any oral frailty groups, along with crude odds ratios (cOR) and adjusted odds ratios (aOR), were summarized in Tables 3, 4, 5 and 6. After adjusting for age, sex and education level, results showed that sedentary time with 5–8 h/d (aOR = 0.54; 95%CI 0.30–0.98) was associated with a decreased likelihood of oral frailty, and passive smoking (aOR = 2.04; 95%CI 1.03–4.03) had an increased risk of having oral frailty by using the OFI-8 scale. With the OFI-8 + TN measurement method, being widowed/unmarried (aOR = 2.53; 95%CI 1.25–5.10) was identified as a risk factor. With the OFI-8 + ODK measurement method, sedentary time with 5-8 h/d (aOR = 0.46; 95%CI 0.26–0.83) and ≥ 8 h/d (aOR = 0.22; 95%CI 0.07–0.74) were shown to be protective factors for oral frailty. Pre-frailty (aOR = 1.76; 95%CI 1.03–3.01) and physical frailty (aOR = 3.01; 95%CI 1.39–6.54) were found to be risk factors for oral frailty. With the measurement method of OFI-8 + TN + ODK, 80 years old and above (cOR = 3.99; 95%CI 1.35–11.81) and being widowed/unmarried (aOR = 2.94; 95%CI 1.12–7.77) were risk factors for oral frailty.

**Table 3:** Factors associated with oral frailty according to the OFI-8

Variables	Model 1 ^*^ OR (95% CI)	P	Model 2 ^†^ OR (95% CI)	P
Marriage				
married	1		1	
widowed/unmarried	1.53 (0.73, 3.22)	0.26	1.78 (0.81, 3.90)	0.15
Employment status				
employed	1		1	
retired	1.29 (0.54, 3.05)	0.57	1.30 (0.54, 3.14)	0.56
others	2.06 (0.63, 6.74)	0.23	2.11 (0.63, 7.10)	0.23
Source of income				
pension	1		1	
child support or others	0.5 (0.24, 1.04)	0.06	0.52 (0.24, 1.11)	0.09
government subsidy	0.87 (0.46, 1.63)	0.66	0.87 (0.45, 1.69)	0.68
Alcohol drinking				
no	1		1	
yes	0.65 (0.34, 1.24)	0.19	0.52 (0.26, 1.05)	0.07
Passive smoking				
no	1		1	
yes	1.79 (0.94, 3.43)	0.08	**2.04 (1.03, 4.03)**	**0.04**
Sedentary time				
<5 h/d	1		1	
5 ~ 8 h/d	0.58 (0.32, 1.03)	0.06	**0.54 (0.30, 0.98)**	**0.044**
≥ 8 h/d	0.47 (0.14, 1.56)	0.22	0.44 (0.13, 1.50)	0.19
Frailty				
robustness	1		1	
pre-frailty	1.37 (0.78, 2.40)	0.27	1.41 (0.80, 2.48)	0.24
frailty	2.23 (0.95, 5.26)	0.07	2.09 (0.88, 4.95)	0.10
Hypertension				
no	1		1	
yes	1.36 (0.81, 2.29)	0.24	1.41 (0.83, 2.40)	0.20
Coronary heart disease				
no	1		1	
yes	2.14 (0.73, 6.27)	0.16	2.08 (0.71, 6.10)	0.18

*Note* OFI-8, Oral Frailty Index-8; OR, odds ratio; CI, confidence interval. ^*^ Values with *P* < 0.2 in the chi-square test were selected in model (1) ^†^ In addition to variables with *P* < 0.2 in the Chi-square test, age, gender, and education were also adjusted in model (2) Significant values are indicated in bold

**Table 4:** Factors associated with oral frailty according to the OFI-8 and TN

Variables	Model 1^*^ OR (95% CI)	P	Model 2 ^†^ OR (95% CI)	P
Marriage				
married	1		1	
widowed/unmarried	**2.52 (1.25, 5.08)**	**0.01**	**2.53 (1.25, 5.10)**	**0.01**
Sex				
female	1		-	-
male	1.65 (0.88, 3.06)	0.12	-	-
Age group (years)				
60–69	1		-	-
70–79	1.73 (0.94, 3.19)	0.08	-	-
80+	2.46 (0.85, 7.1)	0.10	-	-
Employment status				
employed	1		1	
retired	1.44 (0.49, 4.26)	0.51	1.45 (0.49, 4.28)	0.50
others	2.38 (0.67, 8.5)	0.18	2.41 (0.68, 8.60)	0.14
Source of income				
pension	1		1	
child support or others	0.49 (0.19, 1.26)	0.14	0.51 (0.19, 1.32)	0.16
government subsidy	0.89 (0.39, 2.02)	0.78	0.92 (0.40, 2.11)	0.85
Monthly incomes				
<2000	1		1	
2000–4000	0.72 (0.29, 1.74)	0.46	0.69 (0.28, 1.71)	0.42
>4000	0.39 (0.14, 1.11)	0.08	0.38 (0.13, 1.09)	0.07
Smoking				
no	1		1	
yes	1.66 (0.82, 3.37)	0.16	1.66 (0.82, 3.36)	0.16
Rheumatoid arthritis				
no	1		1	
yes	3.61 (0.85, 15.37)	0.08	3.57 (0.84, 15.13)	0.08

*Note* OFI-8, Oral Frailty Index-8; TN, number of natural teeth; OR, odds ratio; CI, confidence interval. ^*^ Values with *P* < 0.2 in the chi-square test were selected in model (1) ^†^ In addition to variables with *P* < 0.2 in the Chi-square test, age, gender, and education were also adjusted in model (2) Significant values are indicated in bold

**Table 5:** Factors associated with oral frailty according to the OFI-8 and ODK

Variables	Model 1^*^ OR (95% CI)	P	Model 2 ^†^ OR (95% CI)	P
Sex				
female	1		-	
male	0.89 (0.53, 1.52)	0.67	-	-
Age group (years)				
60–69	1		-	
70–79	1.06 (0.62, 1.79)	0.84	-	-
80+	2.39 (0.82, 7.02)	0.11	-	-
Employment status				
employed	1		1	
retired	0.97 (0.41, 2.29)	0.94	0.93 (0.39, 2.20)	0.87
others	1.2 (0.4, 3.59)	0.75	1.15 (0.38, 3.43)	0.81
Alcohol drinking				
no	1		1	
yes	0.58 (0.29, 1.13)	0.11	0.57 (0.29, 1.11)	0.10
Sedentary time				
<5 h/d	1		1	
5 ~ 8 h/d	**0.46 (0.26, 0.82)**	**0.009**	**0.46 (0.26, 0.83)**	**0.009**
≥ 8 h/d	**0.23 (0.07, 0.76)**	**0.016**	**0.22 (0.07, 0.74)**	**0.014**
Passive smoking				
no	1		1	
yes	1.32 (0.73, 2.40)	0.36	1.33 (0.73, 2.41)	0.35
Hypertension				
no	1		1	
yes	1.41 (0.86, 2.31)	0.17	1.40 (0.85, 2.29)	0.18
Rheumatoid arthritis				
no	1		1	
yes	6.33 (0.72, 55.49)	0.10	6.54 (0.75, 57.11)	0.09
Diabetes				
no	1		1	
yes	1.74 (0.94, 3.23)	0.08	1.82 (0.99, 3.35)	0.056
Frailty				
robustness	1		1	
pre-frailty	**1.72 (1.01, 2.95)**	**0.046**	**1.76 (1.03, 3.01)**	**0.039**
frailty	**2.94 (1.36, 6.4)**	**0.006**	**3.01 (1.39, 6.54)**	**0.005**

*Note* OFI-8, Oral Frailty Index-8; ODK, Oral diadochokinesis; OR, odds ratio; CI, confidence interval. ^*^ Values with *P* < 0.2 in the chi-square test were selected in model (1) ^†^ In addition to variables with *P* < 0.2 in the Chi-square test, age, gender, and education were also adjusted in model (2) Significant values are indicated in bold

**Table 6:** Factors associated with oral frailty according to the OFI-8, TN and ODK

Variables	Model 1 ^*^ OR (95% CI)	P	Model 2 ^†^ OR (95% CI)	P
Age group (years)				
60–69	1		-	
70–79	1.90 (0.98, 3.71)	0.06	-	-
80+	**3.99 (1.35, 11.81)**	**0.012**	-	-
Marriage				
married	1		1	
widowed/unmarried	**2.75 (1.05, 7.18)**	**0.039**	**2.94 (1.12, 7.77)**	**0.029**
Employment status				
employed	1			
retired	1.19 (0.38, 3.75)	0.77	1.23 (0.39, 3.91)	0.73
others	1.71 (0.46, 6.46)	0.43	1.77 (0.47, 6.74)	0.40
Living arrangement				
living alone	1		1	
with spouse	2.49 (0.91, 6.77)	0.07	2.43 (0.89, 6.65)	0.09
with children	2.56 (0.84, 7.87)	0.10	2.61 (0.85, 8.05)	0.10
others	1.08 (0.18, 6.55)	0.93	1.07 (0.18, 6.43)	0.94
Passive smoking				
no	1		1	
yes	1.60 (0.80, 3.22)	0.19	1.70 (0.83, 3.47)	0.15
Rheumatoid arthritis				
no				
yes	3.65 (0.83, 16.01)	0.09	3.76 (0.86, 16.51)	0.08
Diabetes				
no	1		1	
yes	1.61 (0.81, 3.19)	0.18	1.63 (0.82, 3.23)	0.16

*Note* OFI-8, Oral Frailty Index-8; TN, number of natural teeth; ODK, Oral diadochokinesis; OR, odds ratio; CI, confidence interval. ^*^ Values with *P* < 0.2 in the chi-square test were selected in model (1) ^†^ In addition to variables with *P* < 0.2 in the Chi-square test, age, gender, and education were also adjusted in model (2) Significant values are indicated in bold

### Sensitivity analysis

The sensitivity analysis, as shown in Supplementary Tables 1–4, indicated that results of sedentary time with 5-8 h/d with the OFI-8 scale, being widowed/unmarried with the OFI-8 + TN measurement method, sedentary time with 5-8 h/d, pre-frailty and physical frailty by using the OFI-8 + ODK measurement method and being widowed/unmarried with the OFI-8 + TN + ODK measurement method did not change the findings. Interestingly, passive smoking with the OFI-8 scale was not statistically significantly associated with oral frailty, while living with a spouse became a risk factor for oral frailty in the measurement of the OFI-8 + TN + ODK method.

## Discussion

The wide variation of diagnosis of oral frailty makes it is necessary to examine the prevalence of oral frailty by applying different measurement methods. In this study, we investigated the prevalence of oral frailty by using the OFI-8 scale, natural teeth number, oral diadochokinesis, and repeated saliva swallowing tests. Results showed that the prevalence of oral frailty was much higher by using the OFI-8 scale only compared to combined with other objective measurements. Sedentary time over 5 h per day was a protective factor. Passive smoking, being widowed/unmarried, and physical frailty were found to be risk factors for oral frailty.

### Prevalence of oral frailty

The prevalence of oral frailty by using the OFI-8 scale (69.0%) was higher than other studies which also applied the OFI-8. Tang et al. [8] reported the prevalence of oral frailty in a rural place in China was 44.7% and by using the OFI-8 scale. Interestingly, this is not consistent with previous findings that people in rural areas are more likely to have oral health problems compared to people in urban areas [29,30]. The potential reason may be the item of teeth brushing in Tang’s et al. study was set to be twice per day instead of three times per day, by which more people can meet the requirement. While we followed the original design of the OFI-8 scale by setting the item as brushing the teeth three times per day. This explanation is fulfilled with the results from another study [28] in which the prevalence of oral frailty (33.8%) was lower than in our study, the researchers also set the item as “whether brush your teeth twice per day”.

In addition, the oral diadochokinesis test is sensitive to detect oral frailty as the prevalence of oral frailty by using the OFI-8 + ODK ranked the 2nd highest among all the measurements in this study. ODK is reported to be an important component under the oral function concept which is used to reflect the function of lip and tongue [31]. Especially, ODK is reported to have close associations with swallowing function, the cut-off values were 71 years of age and ODK /pa/ sound 6.2 times/s in Japanese older people ^[[32]]^. Comparatively, the number of teeth is a later symptom shown in oral frail people. Only a small portion of participants showed signs of swallowing dysfunction, which suggested that RSST may be more suitable as a grade rating tool instead of a diagnostic method for oral frailty.

### Risk factors of oral frailty

Pre-frailty and frailty, passive smoking, and being widowed/unmarried were found to be risk factors for oral frailty in this study. The interaction mechanism between oral frailty and physical frailty is interesting to be explored. In previous research, oral frailty is an important reason for poor gait performance, physical frailty, and sarcopenia [1,33]. In this study, people with physical frailty are 3 times more likely to develop oral frailty than robust people, this is consistent with the findings from another cross-sectional study of 589 South Americans, which showed the frequency of physical frailty was 2 times higher among people with oral frailty extremely for older women [34]. Frailty is often associated with muscle weakness and decreased physical activity, which can affect the muscles involved in chewing and swallowing [34]. Additionally, frail individuals may have difficulty maintaining proper oral hygiene and accessing dental care, leading to oral health issues that can contribute to oral frailty. In turn, oral frailty can lead to malnutrition and deteriorate physical frail condition [35].

Another interesting finding in this study is that there was no significant association between smoking and oral frailty, but passive smoking was found to be a risk factor with an OR value of around 2. Massive research with a big sample size has already reported that exposure to smoking had harmful effects on dental health [36,37].

In our study, being widowed/unmarried was also a risk factor for oral frailty, which was consistent with studies investigating relationships between marital status and oral health. Previous studies have shown that being widowed was linked to increased periodontal attachment loss [38], and having fewer sound or filled teeth [39]. These results demonstrated the importance of social relationships in the oral frailty of older adults. Nevertheless, living with a spouse became a risk factor for oral frailty in the sensitivity analysis. Mohamad et al. also found that older persons who lived with a spouse/partner had 1.96 times higher odds of having poor oral health-related quality of life [40], suggesting that social support influenced older people’s oral health-related quality of life. Although the results of sensitivity analyses need to be treated with caution, it is worth us to explore social relationships in the oral frailty of older adults.

In addition, according to Tu’s research, female older people with advanced age and low education level are more likely to have oral frailty [9]. But in the present study, after adjusting the age, sex, and education level, the results showed little to no change except the risk factor analysis by referring to OFI-8 only (Table 3). The potential reason may be more participants were diagnosed with oral frailty according to OFI-8 which occupied a relatively larger sample size compared to by using other measurement methods.

Furthermore, sedentary time over 5 h per day in our study was a protective factor for oral frailty. Raichlen et al. [41] found that not all sedentary behaviors negatively affect cognitive function. Major et al. [42] also found that some types of sedentary behavior may have benefits for cognitive function and highlighted the importance of measuring different domains of sitting time. In our study, the method we chose for measuring sedentary behaviour was based on self-report. Future studies can explore the relationship with oral frailty by measuring the time of different sedentary behaviours.

### Limitations

There are some limitations in this study. Firstly, lifestyle as a covariate relies on self-report and may be subject to recall bias or underreporting. Secondly, the chronic disease variables in the study were not graded by severity, and the effect of chronic disease severity on oral frailty could not be assessed. Thirdly, due to the cross-sectional nature of the study design, we cannot from current evidence determine a causal relationship. Multicenter studies with a larger sample size and longer follow-ups are warranted in the future.

## Conclusions and implications

The prevalence of oral frailty decreased with the combinations of subjective and objective indicators indicating that only subjective assessment was not enough for assessing oral frailty. Among objective indicators, RSST was more suitable to be a grade rating tool for oral frailty instead of being a diagnostic criterion due to the extremely low prevalence. In addition, objective indicators such as TN and ODK should be valued for early screening and preventive interventions. Furthermore, this study helps to identify potential risk factors for oral frailty, which can stimulate health authorities to develop targeted interventions and allocate resources effectively.

Tables.

### Electronic supplementary material

Below is the link to the electronic supplementary material.

Supplementary Material 1

Supplementary Material 2

## Data Availability

No datasets were generated or analysed during the current study.

## Abbreviations

OFI-8: Oral Frailty Index-8 scale
TN: the number of natural teeth
RSST: repetitive saliva-swallowing test
ODK: oral diadochokinesis
GDS-5: 5-item Geriatric Depression Scale
OF: oral frailty

## References

[1] Tanaka T, Takahashi K, Hirano H, Kikutani T, Watanabe Y, Ohara Y (2018). Oral Frailty as a risk factor for physical Frailty and Mortality in Community-Dwelling Elderly. J Gerontol Biol Sci Med Sci.

[2] Dibello V, Zupo R, Sardone R, Lozupone M, Castellana F, Dibello A (2021). Oral frailty and its determinants in older age: a systematic review. Lancet Healthy Longev.

[3] Iwasaki M, Motokawa K, Watanabe Y, Shirobe M, Inagaki H, Edahiro A, et al. A two-year longitudinal study of the Association between oral Frailty and deteriorating Nutritional Status among Community-Dwelling older adults. Int J Environ Res Public Health. 2020;18(1). 10.3390/ijerph18010213. 10.3390/ijerph18010213 PMC7796237 33396639

[4] Parisius KGH, Wartewig E, Schoonmade LJ, Aarab G, Gobbens R, Lobbezoo F (2022). Oral frailty dissected and conceptualized: a scoping review. Arch Gerontol Geriatr.

[5] Hakeem FF, Bernabé E, Sabbah W (2019). Association between oral health and frailty: a systematic review of longitudinal studies. Gerodontology.

[6] Hironaka S, Kugimiya Y, Watanabe Y, Motokawa K, Hirano H, Kawai H (2020). Association between oral, social, and physical frailty in community-dwelling older adults. Arch Gerontol Geriatr.

[7] Komatsu R, Nagai K, Hasegawa Y, Okuda K, Okinaka Y, Wada Y, et al. Association between Physical Frailty subdomains and oral Frailty in Community-Dwelling older adults. Int J Environ Res Public Health. 2021;18(6). 10.3390/ijerph18062931. 10.3390/ijerph18062931 PMC8001836 33809322

[8] Ji T, Xiaoyan T, Li Z, Hao C, Xing Y, Quanxiang Z (2023). Prevalence and influencing factors of oral frailty in the elderly of rural areas in Guizhou Province. Chin J Prev Control Chronic Dis.

[9] Nagatani M, Tanaka T, Son BK, Kawamura J, Tagomori J, Hirano H (2023). Oral frailty as a risk factor for mild cognitive impairment in community-dwelling older adults: Kashiwa study. Exp Gerontol.

[10] Kusunoki H, Ekawa K, Kato N, Yamasaki K, Motone M, Shinmura K (2023). Association between oral frailty and cystatin C-related indices-A questionnaire (OFI-8) study in general internal medicine practice. PLoS ONE.

[11] Hidaka R, Masuda Y, Ogawa K, Tanaka T, Kanazawa M, Suzuki K (2023). Impact of the Comprehensive Awareness Modification of Mouth, Chewing and Meal (CAMCAM) Program on the attitude and behavior towards oral Health and Eating habits as Well as the Condition of oral Frailty: a pilot study. J Nutr Health Aging.

[12] Yang C, Gao Y, An R, Lan Y, Yang Y, Wan Q (2024). Oral frailty: a concept analysis. J Adv Nurs.

[13] Dibello V, Lobbezoo F, Lozupone M, Sardone R, Ballini A, Berardino G (2023). Oral frailty indicators to target major adverse health-related outcomes in older age: a systematic review. Geroscience.

[14] TU H, ZHANG S, Fang Y, He G (2023). Current situation and influencing factors of oral frailty in the community elderly. Chin J Nurs.

[15] Kosaka S, Ohara Y, Naito S, Iimori S, Kado H, Hatta T (2020). Association among kidney function, frailty, and oral function in patients with chronic kidney disease: a cross-sectional study. BMC Nephrol.

[16] Calzada MT, Posada-López A, Gutiérrez-Quiceno B, Botero JE (2021). Association between Tobacco Smoking, Dental Status and Self-perceived oral health in Elderly adults in Colombia. J Cross Cult Gerontol.

[17] Islas-Granillo H, Borges-Yañez SA, Navarrete-Hernández JJ, Veras-Hernández MA, Casanova-Rosado JF, Minaya-Sánchez M (2019). Indicators of oral health in older adults with and without the presence of multimorbidity: a cross-sectional study. Clin Interv Aging.

[18] Petersen PE, Baez RJ, Ogawa H (2020). Global application of oral disease prevention and health promotion as measured 10 years after the 2007 World Health Assembly statement on oral health. Community Dent Oral Epidemiol.

[19] von Elm E, Altman DG, Egger M, Pocock SJ, Gøtzsche PC, Vandenbroucke JP (2007). The strengthening the reporting of Observational studies in Epidemiology (STROBE) statement: guidelines for reporting observational studies. Lancet.

[20] Morris JC (1993). The clinical dementia rating (CDR): current version and scoring rules. Neurology.

[21] Tanaka T, Hirano H, Ohara Y, Nishimoto M, Iijima K (2021). Oral Frailty Index-8 in the risk assessment of new-onset oral frailty and functional disability among community-dwelling older adults. Arch Gerontol Geriatr.

[22] Shiozu H, Higashijima M, Koga T (2015). Association of Sarcopenia with swallowing problems, related to nutrition and activities of daily living of elderly individuals. J Phys Ther Sci.

[23] Ziegler W (2002). Task-related factors in oral motor control: speech and oral diadochokinesis in dysarthria and apraxia of speech. Brain Lang.

[24] Hoyl MT, Alessi CA, Harker JO, Josephson KR, Pietruszka FM, Koelfgen M (1999). Development and testing of a five-item version of the geriatric Depression Scale. J Am Geriatr Soc.

[25] Fried LP, Tangen CM, Walston J, Newman AB, Hirsch C, Gottdiener J (2001). Frailty in older adults: evidence for a phenotype. J Gerontol Biol Sci Med Sci.

[26] Sato T, Hanyu H, Koyama Y, Horita H, Aoki T, Hirao K (2021). Discrepancy between the degree of cognitive impairment and brain imaging abnormalities in Alzheimer’s Disease patients is Associated with Cognitive Reserve. J Alzheimers Dis.

[27] Iwasaki M, Watanabe Y, Motokawa K, Shirobe M, Inagaki H, Motohashi Y (2021). Oral frailty and gait performance in community-dwelling older adults: findings from the Takashimadaira study. J Prosthodont Res.

[28] TU H, Zhang S, Fang Y, He G (2023). Current situation and influencing factors of oral frailty in the community elderly. Chin J Nurs.

[29] Chen HF, Lin YT, Lin JY, Lee HE (2023). Rural-urban disparities in oral health-related quality of life for middle-aged and older adults with diabetes in Taiwan. Front Public Health.

[30] Pan American Health Organization (2023). Oral health status of older adults in the Americas.

[31] Schimmel M, Domioni T, Bukvic H, Arakawa I, Seifert E, Abou-Ayash S (2022). Oral diadochokinesis and associated oro-facial function in young and old German mother-tongue speakers: a cross-sectional study. Gerodontology.

[32] Takeuchi N, Sawada N, Ekuni D, Morita M (2021). Oral diadochokinesis is related to decline in swallowing function among community-dwelling Japanese elderly: a cross-sectional study. Aging Clin Exp Res.

[33] Kugimiya Y, Iwasaki M, Ohara Y, Motokawa K, Edahiro A, Shirobe M, et al. Relationship between oral hypofunction and Sarcopenia in Community-Dwelling older adults: the Otassha Study. Int J Environ Res Public Health. 2021;18(12). 10.3390/ijerph18126666. 10.3390/ijerph18126666 PMC8296410 34205795

[34] Cruz-Moreira K, Alvarez-Cordova L, González-Palacios Torres C, Chedraui P, Jouvin J, Jiménez-Moleón JJ (2023). Prevalence of frailty and its association with oral hypofunction in older adults: a gender perspective. BMC Oral Health.

[35] Tani A, Mizutani S, Oku S, Yatsugi H, Chu T, Liu X (2022). Association between oral function and physical pre-frailty in community-dwelling older people: a cross-sectional study. BMC Geriatr.

[36] Kim YR, Jang KA. Differences in oral health and generalized anxiety disorder according to secondhand smoke exposure in Public places. Behav Sci (Basel). 2023;13(6). 10.3390/bs13060455. 10.3390/bs13060455 PMC10295206 37366707

[37] Uthayakumar T, Bennett JX, Cartas HL, Brunet M, Vo KL, Kroon J (2023). Passive smoking and Oral Health of Infants, preschoolers, and children: a systematic review. Nicotine Tob Res.

[38] Sabbah W, Tsakos G, Chandola T, Newton T, Kawachi I, Sheiham A (2011). The relationship between social network, social support and periodontal disease among older americans. J Clin Periodontol.

[39] Tsakos G, Sabbah W, Chandola T, Newton T, Kawachi I, Aida J (2013). Social relationships and oral health among adults aged 60 years or older. Psychosom Med.

[40] Mohamad Fuad MA, Yacob H, Mohamed N, Wong NI (2020). Association of sociodemographic factors and self-perception of health status on oral health-related quality of life among the older persons in Malaysia. Geriatr Gerontol Int.

[41] Raichlen DA, Klimentidis YC, Sayre MK, Bharadwaj PK, Lai MHC, Wilcox RR (2022). Leisure-time sedentary behaviors are differentially associated with all-cause dementia regardless of engagement in physical activity. Proc Natl Acad Sci U S A.

[42] Major L, Simonsick EM, Napolitano MA, DiPietro L (2023). Domains of sedentary behavior and cognitive function: the Health, Aging, and body composition study, 1999/2000 to 2006/2007. J Gerontol Biol Sci Med Sci.

