# Supplementary material for: Epidemiology and risk factors of oral frailty among older people: an observational study from China
Source: BMC Oral Health. 2024 Mar 21;24:368. doi: 10.1186/s12903-024-04149-1 (PMC10958975; doi:10.1186/s12903-024-04149-1)
Supplement: Supplementary file 2 — Supplementary Material 2 [file 12903_2024_4149_MOESM2_ESM.docx]

**Supplementary Table 1. Factors Associated with Oral Frailty According to the OFI-8**

| **Variables** | **Model 1 ^＊^**  OR (95% CI) | ***P*** | **Model 2 ^†^**  OR (95% CI) | ***P*** |
| --- | --- | --- | --- | --- |
| Marriage |  |  |  |  |
| married | 1 |  | 1 |  |
| widowed/unmarried | 1.51 (0.73, 3.12) | 0.27 | 1.73 (0.81, 3.68) | 0.16 |
| Employment status |  |  |  |  |
| employed | 1 |  | 1 |  |
| retired | 1.36 (0.61, 3.02) | 0.45 | 1.38 (0.61, 3.13) | 0.43 |
| others | 2.00 (0.69, 5.84) | 0.20 | 2.12 (0.72, 6.28) | 0.18 |
| Source of income |  |  |  |  |
| pension | 1 |  | 1 |  |
| child support or others | 0.69 (0.35, 1.35) | 0.28 | 0.72 (0.36, 1.44) | 0.35 |
| government subsidy | 1.01 (0.56, 1.85) | 0.97 | 1.01 (0.54, 1.90) | 0.98 |
| Alcohol drinking |  |  |  |  |
| no | 1 |  | 1 |  |
| yes | 0.75 (0.40, 1.39) | 0.36 | 0.63 (0.33, 1.22) | 0.17 |
| Passive smoking |  |  |  |  |
| no | 1 |  | 1 |  |
| yes | 1.49 (0.81, 2.75) | 0.20 | 1.63 (0.86, 3.08) | 0.13 |
| Sedentary time |  |  |  |  |
| ＜5h/d | 1 |  | 1 |  |
| 5~8h/d | 0.60 (0.35, 1.04) | 0.07 | **0.57 (0.33, 0.99)** | **0.047** |
| ≥8h/d | 0.78 (0.25, 2.43) | 0.67 | 0.75 (0.24, 2.35) | 0.62 |
| Frailty |  |  |  |  |
| robustness | 1 |  | 1 |  |
| pre-frailty | 1.42 (0.83, 2.42) | 0.20 | 1.44 (0.84, 2.46) | 0.19 |
| frailty | 1.78 (0.81, 3.92) | 0.15 | 1.68 (0.76, 3.73) | 0.20 |
| Hypertension |  |  |  |  |
| no | 1 |  | 1 |  |
| yes | 1.24 (0.76, 2.02) | 0.40 | 1.26 (0.77, 2.07) | 0.37 |
| Coronary heart disease |  |  |  |  |
| no | 1 |  | 1 |  |
| yes | 2.24 (0.79, 6.31) | 0.13 | 2.16 (0.77, 6.10) | 0.15 |

Note. OFI-8, Oral Frailty Index-8; OR, odds ratio; CI, confidence interval. ^＊^ Values with *P* < 0.2 in the chi-square test were selected in model 1. ^†^ In addition to variables with *P* < 0.2 in the Chi-square test, age, gender, and education were also adjusted in model 2. Significant values are indicated in bold.

**Supplementary Table 2. Factors Associated with Oral Frailty According to the OFI-8 and TN**

| **Variables** | **Model 1 ^＊^**  OR (95% CI) | ***P*** | **Model 2 ^†^**  OR (95% CI) | ***P*** |
| --- | --- | --- | --- | --- |
| Marriage |  |  |  |  |
| married | 1 |  | 1 |  |
| widowed/unmarried | **2.61 (1.32, 5.18)** | **0.006** | **2.61 (1.32, 5.18)** | **0.006** |
| Sex |  |  |  |  |
| female | 1 |  | - | - |
| male | 1.78 (0.98, 3.24) | 0.06 | - | - |
| Age group (years) |  |  |  |  |
| 60-69 | 1 |  | - | - |
| 70-79 | **1.87 (1.04, 3.34)** | **0.036** | - | - |
| 80+ | 2.32 (0.83, 6.55) | 0.11 | - | - |
| Employment status |  |  |  |  |
| employed | 1 |  | 1 |  |
| retired | 1.34 (0.49, 3.63) | 0.57 | 1.36 (0.5, 3.69) | 0.54 |
| others | 1.88 (0.58, 6.14) | 0.30 | 1.91 (0.59, 6.22) | 0.28 |
| Source of income |  |  |  |  |
| pension | 1 |  | 1 |  |
| child support or others | 0.46 (0.19, 1.13) | 0.09 | 0.48 (0.2, 1.15) | 0.10 |
| government subsidy | 0.92 (0.42, 2.02) | 0.84 | 0.94 (0.43, 2.05) | 0.88 |
| Monthly incomes |  |  |  |  |
| ＜2000 | 1 |  | 1 |  |
| 2000-4000 | 0.74 (0.33, 1.69) | 0.48 | 0.75 (0.33, 1.7) | 0.48 |
| ＞4000 | 0.4 (0.15, 1.06) | 0.07 | 0.4 (0.15, 1.05) | 0.06 |
| Smoking |  |  |  |  |
| no | 1 |  | 1 |  |
| yes | 1.74 (0.88, 3.44) | 0.11 | 1.75 (0.88, 3.46) | 0.11 |
| Rheumatoid arthritis |  |  |  |  |
| no | 1 |  | 1 |  |
| yes | 3.4 (0.84, 13.72) | 0.086 | 3.33 (0.83, 13.36) | 0.09 |

Note. OFI-8, Oral Frailty Index-8; TN, number of natural teeth; OR, odds ratio; CI, confidence interval. ^＊^ Values with *P* < 0.2 in the chi-square test were selected in model 1. ^†^ In addition to variables with *P* < 0.2 in the Chi-square test, age, gender, and education were also adjusted in model 2. Significant values are indicated in bold.

**Supplementary Table 3. Factors Associated with Oral Frailty According to the OFI-8 and ODK**

| **Variables** | **Model 1 ^＊^**  OR (95% CI) | ***P*** | **Model 2 ^†^**  OR (95% CI) | ***P*** |
| --- | --- | --- | --- | --- |
| Sex |  |  |  |  |
| female | 1 |  | - |  |
| male | 0.85 (0.52, 1.38) | 0.504 | - | - |
| Age group (years) |  |  |  |  |
| 60-69 | 1 |  | - |  |
| 70-79 | 1.07 (0.66, 1.76) | 0.78 | - | - |
| 80+ | 1.71 (0.63, 4.67) | 0.30 | - | - |
| Employment status |  |  |  |  |
| employed | 1 |  | 1 |  |
| retired | 1.18 (0.53, 2.62) | 0.69 | 1.15 (0.52, 2.57) | 0.73 |
| others | 1.54 (0.57, 4.17) | 0.40 | 1.51 (0.55, 4.10) | 0.42 |
| Alcohol drinking |  |  |  |  |
| no | 1 |  | 1 |  |
| yes | 0.68 (0.36, 1.27) | 0.23 | 0.67 (0.36, 1.25) | 0.21 |
| Sedentary time |  |  |  |  |
| ＜5h/d | 1 |  | 1 |  |
| 5~8h/d | **0.53 (0.31, 0.9)** | **0.019** | **0.53 (0.31, 0.9)** | **0.019** |
| ≥8h/d | 0.47 (0.17, 1.28) | 0.14 | 0.46 (0.17, 1.26) | 0.13 |
| Passive smoking |  |  |  |  |
| no | 1 |  | 1 |  |
| yes | 1.13 (0.64, 1.97) | 0.68 | 1.13 (0.64, 1.97) | 0.68 |
| Hypertension |  |  |  |  |
| no | 1 |  | 1 |  |
| yes | 1.15 (0.73, 1.83) | 0.54 | 1.15 (0.73, 1.83) | 0.54 |
| Rheumatoid arthritis |  |  |  |  |
| no | 1 |  | 1 |  |
| yes | 6.25 (0.75, 51.73) | 0.09 | 6.31 (0.76, 52.25) | 0.09 |
| Diabetes |  |  |  |  |
| no | 1 |  | 1 |  |
| yes | 1.68 (0.92, 3.07) | 0.09 | 1.72 (0.94, 3.12) | 0.08 |
| Frailty |  |  |  |  |
| robustness | 1 |  | 1 |  |
| pre-frailty | **1.66 (1.01, 2.75)** | **0.047** | **1.67 (1.01, 2.75)** | **0.046** |
| frailty | **2.44 (1.19, 5.01)** | **0.015** | **2.46 (1.20, 5.05)** | **0.014** |

Note. OFI-8, Oral Frailty Index-8; ODK, Oral diadochokinesis; OR, odds ratio; CI, confidence interval. ^＊^ Values with *P* < 0.2 in the chi-square test were selected in model 1. ^†^ In addition to variables with *P* < 0.2 in the Chi-square test, age, gender, and education were also adjusted in model 2. Significant values are indicated in bold. The *P* value of the Hosmer and Lemeshow Test after adjusting for age, gender, and education was less than 0.05, which indicates that the logistics regression model has a poor fit.

**Supplementary Table 4. Factors Associated with Oral Frailty According to the OFI-8, TN and ODK**

| **Variables** | **Model 1 ^＊^**  OR (95% CI) | ***P*** | **Model 2 ^†^**  OR (95% CI) | ***P*** |
| --- | --- | --- | --- | --- |
| Age group (years) |  |  |  |  |
| 60-69 | 1 |  | - |  |
| 70-79 | **2.18 (1.16, 4.10)** | **0.015** | - | - |
| 80+ | **3.91 (1.36, 11.25)** | **0.011** | - | - |
| Marriage |  |  |  |  |
| married | 1 |  | 1 |  |
| widowed/unmarried | **2.98 (1.16, 7.63)** | **0.023** | **3.31 (1.27, 8.61)** | **0.014** |
| Employment status |  |  |  |  |
| employed | 1 |  | 1 |  |
| retired | 1.15 (0.40, 3.27) | 0.80 | 1.21 (0.42, 3.47) | 0.73 |
| others | 1.31 (0.38, 4.46) | 0.67 | 1.38 (0.40, 4.76) | 0.61 |
| Living arrangement |  |  |  |  |
| living alone | 1 |  | 1 |  |
| with spouse | **2.97 (1.12, 7.90)** | **0.029** | **2.89 (1.08, 7.76)** | **0.035** |
| with children | 2.73 (0.92, 8.07) | 0.07 | 2.89 (0.97, 8.65) | 0.06 |
| others | 1.13 (0.19, 6.69) | 0.90 | 1.13 (0.19, 6.70) | 0.89 |
| Passive smoking |  |  |  |  |
| no | 1 |  | 1 |  |
| yes | 1.78 (0.92, 3.43) | 0.09 | 1.91 (0.97, 3.73) | 0.06 |
| Rheumatoid arthritis |  |  |  |  |
| no | 1 |  | 1 |  |
| yes | 3.19 (0.79, 12.99) | 0.11 | 3.37 (0.83, 13.74) | 0.09 |
| Diabetes |  |  |  |  |
| no | 1 |  | 1 |  |
| yes | 1.53 (0.78, 2.99) | 0.22 | 1.50 (0.77, 2.94) | 0.24 |

Note. OFI-8, Oral Frailty Index-8; TN, number of natural teeth; ODK, Oral diadochokinesis; OR, odds ratio; CI, confidence interval. ^＊^ Values with *P* < 0.2 in the chi-square test were selected in model 1. ^†^ In addition to variables with *P* < 0.2 in the Chi-square test, age, gender, and education were also adjusted in model 2. Significant values are indicated in bold.
